# Exploring the role of an unsanctioned, supervised peer driven injection facility in reducing HIV and hepatitis C infections in people that require assistance during injection

**DOI:** 10.1186/s40352-015-0028-0

**Published:** 2015-08-28

**Authors:** Ehsan Jozaghi, Vancouver Area Network of Drug Users

**Affiliations:** School of Criminology, Simon Fraser University, Burnaby, British Columbia Canada

## Abstract

**Background:**

Supervised consumption rooms or supervised injection facilities (SIFs) are venues that have reduced the risk of needle sharing and deaths caused by drug overdose among people who inject drugs (PWID). As a result of such a decline in the mortality rate, numerous studies have been able to illustrate its cost-effectiveness. However, studies have neglected to examine the same phenomena for unsanctioned SIFs that are run by peer drug users and provide assisted injections.

**Methods:**

The current study will determine whether the former unsanctioned SIF, that provided assisted injection and was operated by the grass root organization called Vancouver Area Network of Drug Users (VANDU), cost less than the health care consequences of not having such a program in Vancouver, Canada. By analyzing data gathered in 2013, this paper relies on two mathematical models to estimate the number of new HIV and HCV infections prevented by the former unsanctioned facility in Vancouver’s Downtown Eastside.

**Results:**

A conservative estimate indicates that the SIF location that provided assisted injections has a benefit-cost ratio of 33.1:1 due to its low operational cost. At the baseline sharing rate, the facility, on an average, reduced 81 HCV and 30 HIV cases among PWID each year. Such reductions in blood borne infections among PWID resulted in annual savings worth CAN$4.3 million dollars in health care expenditure. In addition to this, the current paper relies on a sensitivity analysis based on different needle sharing rate scenarios.

**Conclusions:**

The sensitivity analysis and the baseline rates indicate that funding SIF facilities operated by peer drug users that facilitate assisted injection appear to be an efficient and effective use of financial resources in the public health domain since they lead to a significant decline in the rate of mortality within a vulnerable population.

## Background

The neighbourhood that consists of the ten blocks that comprise the Downtown Eastside (DTES) of Vancouver is the most impoverished urban area in Canada – one that is marked with high rates of homelessness, drug abuse, mental illness, and survival sex work [40, 67, 73, 78]. An estimated number of 5,000 people who inject drugs (PWID) call this Vancouver neighbourhood their home [46]. The lack of availability of services for the PWID population concentrated in this area has contributed to the prevalence of human immunodeficiency virus (HIV) and hepatitis C virus (HCV) to an extent that rivals its presence in developing countries during the late 1990s—with cases of overdose similarly widespread [13, 40].

North America’s first and only government sanctioned supervised injection facility (SIF), Insite, opened in September 2003 to combat the growing public health crisis facing the DTES [75, 77]. The public health benefits of the facility in the DTES have been well documented in over 60 peer reviewed studies. However, the facility has been operating at full capacity over the past decade and long line-ups discourage many PWID from visiting the small facility especially during the welfare week, when people on social assistance receive their government issued cheques ([65], p. 565).

There are now more than 92 SIFs operating across the globe, and the potential of expanding Insite in the DTES has been reviewed and endorsed in quite a few peer reviewed studies [26, 48, 51, 65]. In addition to this, a cost-benefit/cost-effectiveness study has suggested that expanding the facility beyond its current location saves tax payers’ money in terms of preventing new cases of HIV in the DTES [4].

However, the Canadian conservative government’s aggressive war on drugs, which has increased law enforcement expenditure while simultaneously steering money away from investment in harm reduction initiatives, has raised questions regarding the efficiency of SIFs in the media as well as in court [16, 25, 71, 72]. Furthermore, the current Canadian conservative government is very unlikely to approve new SIFs in Vancouver despite the proven track record of such facilities across Europe and, close to home, in Vancouver [22]. As numerous studies in North America and Europe have highlighted, PWID are not a homogenous group. Essentially, PWID have unique needs that the current SIF operating in the DTES is not fit to meet. Insite, North America’s only legally sanctioned SIF, currently forbids sharing of drugs, assisted injection and requires registration of clients [38]. Such noted restrictions, in addition to the high volume of usage by PWID and lack of resources for people who smoke drugs (*e.g.*, crack and methamphetamine), discourage many drug users to visit the small facility in the DTES.

Consequently, there is a pressing need for additional SIFs that can accommodate various groups of PWID in the DTES. In response to the general lack of public health interventions, by the federal and provincial government, for PWID in the DTES, a grass root organization known as VANDU (Vancouver Area network of drug users) was formed in the late 1990s [27]. It composed of former and current drug users [38]. VANDU, through activism and social justice initiatives, has been instrumental in changing the harm reduction approach in the city of Vancouver [38].

Through their peer mobilization, and in an effort to prevent the ongoing spread of HIV, HCV and overdose in their community, VANDU opened an unsanctioned SIF only a few blocks from Insite [51]. The small unsanctioned SIF, which had three tables and six chairs, was operated by peers and was free to access. The facility could accommodate 6 potential PWID at the same time. PWID that used the facility were provided with free sterile drug use equipment (*e.g.*, insulin syringes with attached needles, bottles of sterile water for injection, latex condoms, alcohol swaps, disposal boxes and spoons), water, emergency care (in case of overdose), and referral to addiction services by VANDU.

Moreover, the above mentioned unsanctioned SIF allowed the peer drug users who operated the facility to engage in assisted injection – something that is not permitted under the mandates of Insite [51]. In effect, it is estimated that 40 % of PWID require some sort of assistance when injecting in the DTES [57]. This need to rely on others to help with injection has placed many users at increased risk of violence and drug related harm [47, 51]. Furthermore, there are numerous studies that indicate that drug users who require help injecting drugs, when compared to those who self-inject, are at greater risk of HIV and HCV infections as well as more prone to indulge in the harmful practices of needle sharing and drug overdose [35, 36, 43, 55, 57, 63, 76].

Assisted injection at VANDU’s former facility was only permitted under auspices of strict harm reduction practices that prohibited sharing of drugs and injection paraphernalia [51]. Moreover, the peers that operated the injection room were required to have CPR and Naloxone training. Furthermore, the operators of the injection room adopted certain precautionary measures: they were expected to “wear latex gloves, disinfect the injection site, and discard syringes in sharps containers” after use ([51], p. 476). The unsanctioned SIF, however, was forced to shut down in December 2013, when Vancouver Coastal Health threatened to cut off all funding to VANDU’s operating budget of CAN$200,000 [to convert the Canadian to American dollar simply multiply by 0.81] unless the illegal facility ceased to operate.

Two qualitative studies have demonstrated the effectiveness of the facilities in reducing risk factors for blood borne infections “and other health risks, while allowing people who require help injecting to escape drug scene violence” ([51], p. 473; see also [52]). Numerous other research has demonstrated the cost-effectiveness of operating the only sanctioned SIF in North America [3, 5, 10]. Furthermore, the operation and expansion of potential sanctioned SIFs in numerous Canadian jurisdictions, such as Montreal, Ottawa, Saskatoon, Toronto, and Victoria, has been found to be extremely cost-effective [30–33]. However, the concept of an unsanctioned, peer-run SIF is controversial, particularly because the potential cost-effectiveness impacts and benefits of assisted injections are unknown.

Therefore, this study was conducted to examine the effectiveness of an unsanctioned peer-run SIF, wherein peers are known to be actively involved not only in running the facility, but also in overseeing assisted injection practices. The analysis used mathematical models with conservative parameters to estimate the number of HCV and HIV infections prevented as a result of an unsanctioned SIF. The savings from the illnesses avoided were compared to the operational cost of the unsanctioned facility, and then extended to consider the impact of opening additional unsanctioned SIFs of the same size.

## Methods

### Models

This study was approved by both the Simon Fraser University Research Ethics Board and VANDU’s Executive Board. The data collected pertains to the number of visits to the unsanctioned SIF, determined to be 8,886 per year, with an average of 657 per month. For the current analysis, it was necessary to rely on a model that could reflect the effects of providing clean equipment and adopting safer injection behaviors in its calculations. Drawing from the methodology of research on the economic impact of a needle exchange program in Edmonton, the current study uses a mathematical model to estimate the number of HCV and HIV infections that could be prevented through the establishment of an SIF [24]. The number of new HIV/HCV infections avoided, (A), is calculated as follows:

1 $$ \mathrm{New}\kern0.5em \mathrm{H}\mathrm{I}\mathrm{V}/\mathrm{H}\mathrm{C}\mathrm{V}\kern0.5em \mathrm{infections}\kern0.5em (A)= INsd\left[1-{\left(1- qt\right)}^m\right] $$

where (*I*) is the PWID population that is HIV or HCV negative, (*N*) is the number of needles in circulation, (*s*) is the rates of needle sharing, (*d*) is the percentage of needles not cleaned before use, (*q*) is the HCV/HIV prevalence in the PWID population, (*t*) is the probability of HCV/HIV transmission when using an HIV/HCV infected needle, and (*m*) is the number of sharing partners when needles are shared.

The model above has been adopted previously by six costing studies [4, 28, 30–33]. To operationalize the model, the data shown in Table 1 needs to be imputed in excel programing and combined with the reduction in the rate of needle sharing rates in order to calculate the rate of blood borne infection reductions. The current study also relies on a second model to calculate the number of HIV infections avoided:

**Table 1:** Sources for variables used in mathematical modeling

Variable	Value	Source
Proportion of PWID HIV- (I)	77.46 %	Petrar *et al.* [58]); Tyndall *et al.* [69]
Rate of Needle sharing (b) or (λ)	30 %	Holtgrave *et al.* [20]; Kerr *et al.* [37, 39]
Number of needles in circulation (N)	2000000	McClean [50]; Buxton [8]
Percentage of needles not cleaned (d)	17.00 %	Hope *et al.* [21]; Kaplan and O’Keefe [34]; Jacobs *et al.* [24]
Probability of HIV infections from a single injection (t) or (α)	0.67 %	Allard [1]; Kaplan and O’Keefe [34]
Number of sharing partners (m)	1.38	Kozal *et al.* [41]; Jacobs *et al.* [24]
Proportion of PWID HIV+ (q)	22.54 %	Holtgrave *et al.* [20]; Kerr *et al.* [37, 39]
Proportion PWID HCV- (I)	12.00 %	Bayoumi & Zaric [5]
Proportion of PWID HCV+ (q)	88.00 %	Bayoumi & Zaric [5]
Probability of HCV infection from single injection(t)	3 %	Gore & Bird [15]
Proportion of HIV/HCV infected needles (β)	40.50 %	Hope *et al.* [21]; Kaplan and O’Keefe [34])
Probability of needles cleaned (θ)	83 %	Iguchi & Bux [23]; Anderson *et al.* [2]; Kaplan and O’Keefe [34]); Jacobs *et al.* [24]

2 $$ \mathrm{New}\ \mathrm{H}\mathrm{I}\mathrm{V}\ \mathrm{infection}\ \mathrm{rate}\ (A)=\left(1-\pi \right)\lambda \left(1-\theta \right)\beta \alpha $$

where (*β*) is the percentage of HIV infected needles, (*π*) is the prevalence of HIV infections in the neighborhood, (*θ*) is the probability that a borrowed syringe is decontaminated, (*α*) is the probability of acquiring HIV from a single injection with contaminated syringe and (*λ*) is the rate of needle sharing. The second model was used in the evaluation of New Haven needle depot by Kaplan and O’Keefe [34], and subsequently used in Pinkerton [60] Jozaghi *et al.* [31]) and Jozaghi *et al.* [33]. The Kaplan model estimates the rate of infection. Consequently, the rate of HIV infections was calculated with and without the penitential SIF and then multiplied by the number of PWIDs to get the count of HIV infections.

The ratio of cost-effectiveness was estimated by dividing the operational cost of the potential SIF by the number of prevented blood borne infections such as HCV or HIV. An estimation for cost–benefit ratio was reached by dividing the cost savings, in terms of diseases prevented, by the cost of the operations. Furthermore, according to Jozaghi *et al.* [31]), “marginal costs and benefits are measurements for producing one more unit. In contrast, the cumulative benefits and cost are the running total of benefit and cost with each successive unit” (p. 8). The sensitivity analysis has proven to be a reliable way to control and account for the number of uncertainties that may arise in mathematical models. Similarly, this study relied on two sensitivity analyses to control the number of assumptions considered in this study. The first form of sensitivity analysis was achieved by employing a secondary mathematical model to estimate the potential number of HIV cases that have been prevented by establishing an unsanctioned SIF in the DTES. Later, the rate of needle sharing within the DTES was used to account for the additional uncertainties presented in the model.

### Assumptions

The cost-benefit and cost-effectiveness analysis of this study relied on the number of HIV and HCV infections prevented; nevertheless, there were a handful of parameters to be estimated, including the lifetime costs of treating HIV and HCV infections, new HIV and HCV cases prevented based on each additional facility, and subsequently, the desirable number of facilities. For this research, it was necessary to use a model that reflected the effects of using clean injection equipment and safer injecting behaviours in its calculations. Previous SIF cost studies such as those conducted by Bayoumi and Zaric [5], Pinkerton [60], Pinkerton [61], Andresen and Boyd [3], Andresen and Jozaghi [4], Jozaghi and Jackson [30], Jozaghi *et al.* [31]), Jozaghi *et al.* [32]) and Jozaghi *et al.* [33] have concluded that SIFs are able to prevent the risk of new HIV and HCV cases, since there will be a certain number of known non-shared injections as opposed to shared injections outside of the facility.

This study also chose to employ the behaviour change incorporated, where it was assumed that those PWIDs who attend a SIF are at 0.3 frequency to share needles outside of an injection facility (Bravo *et al.* [7, 37, 39]). The behavioural change is a phenomenon that has been documented in the clientele of SIFs in Canada and Europe, where such PWID are less likely to share their needles once they leave the injection facility (Bravo *et al.* [7, 37, 39]). This rate has been incorporated in Bayoumi and Zaric [5], Bayoumi *et al.* [6], Andresen and Boyd [3], Andresen and Jozaghi [4], Jozaghi [27, 28]), Jozaghi and Jackson [30], Jozaghi *et al.* [31]), Jozaghi *et al.* [32]), and Jozaghi *et al.* [33] because of the empirical evidence shown in Kerr *et al.* [37, 39]) and Bravo *et al.* [7]. Ultimately, similar to Bayoumi and Zaric [5], Bayoumi *et al.* [6], Jozaghi [29], Jozaghi *et al.* [32]) and Jozaghi *et al.* [33], this study considered 50 % of the reported effects of a SIF on needle sharing. An odds ratio of 0.60 was employed in the calculation to account for the lower needle sharing rate for those PWID that visited the unsanctioned facility in the DTES so they could have access to assisted injection.

However, to stay on the conservative side, the odds ratio was only employed twice: for the first and second potential unsanctioned SIF where the facilities would be able to accommodate 12 clients at the same time. The behaviour change on the sharing variable indicates that if a second unsanctioned SIF is established, further behavioural changes may occur if and only if new PWID become users of SIF [4]. If only current users of SIF use the second SIF, simply performing more of their injections at SIF, no further behavioural changes can be assumed. In other words, SIFs will only be attracting returning clientele that will be visiting the SIF more frequently because of availability of assisted injection in the DTES [33].

### Variables and parameters

Except for the rate of utilization at VANDU, the values for both equations were drawn from Vancouver-specific estimates, using scientific and medical literature where city-specific estimates were not available. Fundamentally, the cost-benefit/cost-effectiveness analysis of this study relied on the behavioural change and the number of HCV and HIV infections prevented. Also, the total number of injections in the DTES was calculated by multiplying the total population of PWID users and the estimated number of injections per year (*e.g.*, 5,000 PWID * 913 injections per year) [4, 20, 24, 44, 50].

### The medical cost of new HIV infections

The success of ART (antiretroviral therapy) has not been replicated amongst PWID due to the treatment’s side-effects [17], as well as high rates of treatment discontinuation among the marginalized population [18, 59, 68, 75, 77]. For example, a study by Druyts *et al.* [12] found a threefold risk of death among HIV-infected individuals on ART in neighbourhoods with a high concentration of PWID relative to those with a high concentration of gay men, thereby leading to poorer treatment uptake and adherence among PWID [45]. However, it should be noted that recent research by Meyer *et al.* [54] suggests that PWIDs are at a significant risk in terms of being subject to health disparities for treatment of HIV. This can be attributed mainly due to denial of treatment by health care providers to ART and late start of treatment rather than some of the noted issues related to discontinuation of treatment and non-adherence. Nevertheless, the lifetime cost-savings from each averted case of HIV infection ranges from CAN$174,410 [14], to CAN$ 253,210 [9, 19, 62] and as high as CAN$289,970 with addition of ART [70].

This study utilized the numerical figure of CAN$210,555 – as reported in Pinkerton [60], Andresen and Jozaghi [4], Jozaghi and Jackson [30], Jozaghi *et al.* [31]), Jozaghi *et al.* [32]) and Jozaghi *et al.* [33] – because this analysis anticipated a lower cost of treatment for HIV infections among PWID. This lower estimate was chosen because PWID may experience certain self-imposed societal barriers, making them less likely to take full advantage of public health care, especially the expensive ART program [17, 54, 75, 77].

### The medical cost of a new HCV infection

The cost of treating an HCV infection ranges from CAN$30,000 [71, 72] to CAN$69,188 [49]. A lower number of CAN$20,000 has also been suggested for each completed patient course of treatment [42]. However, the value of CAN$35,143 reported in National Centre in HIV Epidemiology and Clinical Research [56], Jozaghi *et al.* [32], Jozaghi [27, 28], Jozaghi *et al.* [31], and Jozaghi *et al.* [33] is the most appropriate for this research, since the above mentioned studies considered the costs for liver transplant cases, liver failure, and hepatocellular carcinoma. Even so, it is essential to acknowledge that the cost of treating an HCV infection is changing rapidly as a direct consequence of the emergence of more effective treatments that tend to be shorter and significantly more expensive.

### Cost of SIF

This analysis used the fixed operating cost of an existing SIF to estimate the total cost involved. The former facility operated from Monday to Friday from 10-7 pm, and from 4-7 pm on weekends. The staff of the former unsanctioned SIF included volunteers that are provided with small stipends amounting collectively to CAN$47,203 per year. When added to the total cost of the rent and the injection equipment, estimated at CAN$50,000, the total annual operating cost of the facility becomes CAN$97,203.

## Results

This research assessed whether VANDU’s unsanctioned SIF has a net positive effect on the Canadian society, and whether this initiative saves public health care funds by averting new HIV and HCV infections. Therefore, the expenses incurred for operating unsanctioned SIFs were determined using two mathematical models. These predicted the number of new cases of HIV infections prevented based on the sharing rate, which included the impact of behavioural changes in injection activities once outside of the unsanctioned SIF. For HCV infections, only the first mathematical model was used since ‘c’, the proportion of HCV infected needles in the second mathematical model, remains unreported in any published or unpublished research.

As shown in Table 1, numerous published peer-reviewed studies were utilized to complete the analysis.

As expected, according to the data in Tables 2 and 3, increasing the scope of an unsanctioned SIF through site expansion would decrease the overall rate of HIV infections. Based on Table 4, the second mathematical model also predicts a range of 10-32 HIV infections, with marginal HIV cases ranging from 3-10.

**Table 2:** The cumulative annual cost - effectiveness and cost – benefit of unsanctioned SIF in Vancouver using Jacobs *et al.*’s [24] model

Variables	Annual cost of operation	Sharing rate	# of HIV averted	# of HCV averted	Cost-effectiveness ratio HCV	Cost-effectiveness ratio HIV	Benefit-cost ratio HCV	Benefit-cost ratio HIV	Cost-benefit ratio total
One SIF	$97,203	25 %	30	81	$1,200	$3,240	29.3	64.9	94.3
		(33 %, 16 %)	(40, 20)	(103, 59)	($944, $1,648)	($2,430, $4,860)	(37.2, 21.3)	(86.6, 43.3)	(123.8, 64.7)
Two SIFs	$194,406	19 %	59	160	$1,215	$3,295	28.9	63.9	92.8
		(26 %, 13 %)	(79, 38)	(207, 104)	($939, $1,869)	($2,460, $5,116)	(37.4, 18.8)	(85.6, 41.2)	(122.9, 60.0)
Three SIFs	$291,609	18 %	66	179	$1,629	$4,418	21.6	47.7	69.2
		(24 %, 12 %)	(88, 44)	(237, 118)	($1,230, $2,471)	($3,314, $6,628)	(28.6, 14.2)	(63.5, 31.8)	(92.1, 46.0)
Four SIFs	$388,812	16 %	75	202	$1,925	$5,184	18.3	40.6	58.9
		(22 %, 11 %)	(99, 50)	(266, 133)	($1,462, $2,923)	($3,927, $7,776)	(24, 12)	(53.6, 27.1)	(77.7, 39.1)
Five SIFs	$486,015	15 %	84	226	$2,151	$5,785	16.3	36.9	52.7
		(19 %, 10 %)	(113, 56)	(311, 148)	($1,563, $3,284)	($4,301, $8,679)	(22.5, 10.7)	(49, 24.3)	(71.4, 35.0)
Six SIFs	$583,218	13 %	93	251	$2,323	$6,271	15.1	33.6	48.7
		(18 %, 9 %)	(123, 62)	(325, 163)	($1,795, $3,578)	($4,741, $9,407)	(19.6, 9.8)	(44.4, 22.4)	(63.9, 32.2)
Seven SIFs	$680,421	12 %	102	274	$2,483	$6,671	14.2	31.6	45.7
		(15 %, 8 %)	(135, 68)	(370, 177)	($1,839, $3,844)	($5,040, $1,006)	(19.1, 3.51)	(41.8, 21)	(61.0, 30.2)

Note: The numbers in parentheses represent the results of the sensitivity analysis: (40 per cent sharing rate, 20 percent sharing rate)

**Table 3:** The marginal annual cost - effectiveness and cost – benefit of unsanctioned SIF in Vancouver using Jacobs *et al.*’s [24] model

Variables	Annual cost of operation	Sharing rate	# of HIV averted	# of HCV averted	Cost-effectiveness ratio HCV	Cost-effectiveness ratio HIV	Benefit-cost ratio HCV	Benefit-cost ratio HIV	Cost-benefit ratio total
One SIF	$97,203	25 %	30	81	$1,200	$3,240	29.3	64.9	94.3
		(33 %, 16 %)	(40, 20)	(103, 59)	($944, $1,648)	($2,430, $4,860)	(37.2, 21.3)	(86.6, 43.3)	(102.2, 64.7)
Two SIFs	$97,203	19 %	29	79	$1,230	$3,352	28.6	62.8	91..4
		(26 %, 13 %)	(39, 38)	(103, 104)	($944, $1,869)	($2,492, $5,116)	(37.2, 18.8)	(84.5, 41.2)	(121.7, 60.0)
Three SIFs	$97,203	18 %	7	19	$5,115	$13,886	6.9	15.2	22.0
		(24 %, 12 %)	(9, 44)	(29, 118)	($3,352, $2,471)	($10,800, $6,628)	(10.5, 14.2)	(19.5, 31.8)	(25.7, 46.0)
Four SIFs	$97,203	16 %	8	23	$4,226	$12,150	8.3	17.3	25.6
		(22 %, 11 %)	(12, 50)	(29, 133)	($3,352, $2,923)	($8,100, $7,776)	(10.5, 12)	(26, 27.1)	(25.6, 39.1)
Five SIFs	$97,203	15 %	9	24	$4,050	$10,800	8.7	19.5	28.2
		(19 %, 10 %)	(14, 56)	(44, 148)	($2,209, $3,284)	($6,943, $8,679)	(15.9, 10.7)	(30.3, 24.3)	(28.2, 35.0)
Six SIFs	$97,203	13 %	9	25	$3,888	$10,800	9	19.5	28.5
		(18 %, 9 %)	(10, 62)	(14, 163)	($6,943, $3,578)	($9,720, $9,407)	(5.1, 9.8)	(21.6, 22.4)	(26.7, 32.2)
Seven SIFs	$97,203	12 %	8	22	$4,418	$12,150	7.9	17.3	25.3
		(15 %, 8 %)	(12, 68)	(44, 177)	($2,209, $3,844)	($8,100, $1,006)	(15.9, 3.51)	(26, 21)	(33.2, 30.2)

Note: The numbers in parentheses represent the results of the sensitivity analysis: (40 per cent sharing rate, 20 percent sharing rate)

**Table 4:** The cumulative and marginal cost - effectiveness and cost – benefit of unsanctioned SIF in Vancouver using Kaplan and O’Keefe’s [34] model

Variables	Annual cost of operation	Sharing rate	# of HIV averted	Cost-effectiveness ratio HIV	Benefit-cost ratio HIV
One SIF	$97,203	25 %	10	$9,720	21.7
	($97,203)		(10)	($9,720)	(21.7)
Two SIFs	$194,406	19 %	19	$10,232	20.6
	($97,203)		(9)	($10,800)	(19.5)
Three SIFs	$291,609	18 %	21	$13,886	15.2
	($97,203)		(2)	($48,601)	(4.3)
Four SIFs	$388,812	16 %	23	$16,905	12.5
	($97,203)		(3)	($32,401)	(6.5)
Five SIFs	$486,015	15 %	27	$18,000	11.7
	($97,203)		(3)	($32,401)	(6.5)
Six SIFs	$583,218	13 %	30	$19,441	10.8
	($97,203)		(3)	($32,401)	(6.5)
Seven SIFs	$680,421	12 %	32	$21,263	9.9
	($97,203)		(3)	($32,401)	(6.5)

Note: The numbers in parentheses represent the marginal results

Table 4, using the first and second mathematical models, shows a substantial difference in the number of new HIV infections prevented. The first model, based on Tables 2 and 3, predicts 30-102 for HIV and 81-274 for HCV, with the marginal range being much smaller, at 7-30 for HIV and 19-81 for HCV. According to Andresen and Jozaghi [4], this disparity may be attributed to the first model that overestimated the cases of HIV infection. However, both models predict that VANDU’s unsanctioned SIF is cost-effective. For example, according to Table 2, benefit-cost ratio for HIV in the first model ranges from 31.6 to 64.9, and cost-effectiveness for HIV ranges from $3,240 to $6,671. The cumulative annual estimates of averted HCV cases translates into a benefit-cost ratio ranging from 14.2 to 29.3, and cost-effectiveness value ranging from $1,200 to $2,483. In contrast, the marginal estimates of VANDU’s unsanctioned SIF result in a much smaller return, both in its benefit-cost and cost-effectiveness ratios. For instance, the marginal benefit-cost ratio varies from 17.3 to 64.9 for HIV and 7.9 to 29.3 for HCV. The marginal cost-effectiveness value ranges from $3,240 to $12,150 for HIV and from $1,200 to $4,418 for HCV.

Moreover, when the second mathematical model is considered, as outlined in Table 4, averted cases of HIV account for a substantial difference between the economic evaluation of SIFs – especially in terms of the cumulative versus the marginal estimates. For example, according to data in Table 4, the cumulative annual estimates of new HIV cases averted, based on the second model, translate into a benefit-cost ratio ranging from 9.9 to 21.7, and cost-effectiveness value ranging from $9,720 to $21,263. In contrast, the marginal estimates of VANDU’s SIF expansion, based on the the second model, result in a much smaller return, in both benefit-cost and cost-effectiveness ratio. For instance, HIV’s marginal benefit-cost ratio varies from 5.5 to 21.7 and its marginal cost-effectiveness from $9,720 to $32,401.

The results from both mathematical models in this paper demonstrate that VANDU’s unsanctioned SIF establishment in the DTES saves taxpayers’ money. Nevertheless, comparing the overall figures (such as cumulative or marginal) seems to support the earlier assumption that expanding unsanctioned SIFs through unsanctioned practices such as assistance during injection will save money if it prevents even a modest number of HIV and HCV infections per year. Finally, a sensitivity analysis was conducted for the models employed, with differing initial needle-sharing rates (see Tables 2, 3 and 4). As with Andresen and Boyd [3], Andresen and Jozaghi [4], Jozaghi [27, 28], Jozaghi and Jackson [30], Jozaghi *et al.* [32], Jozaghi *et al.* [31], and Jozaghi *et al.* [33], the current analysis used sharing rate deviations for the sensitivity analysis; 40 and 20 per cent initial needle-sharing rates were used to account for uncertainties in the models. The results from both the baseline and sensitivity analyses demonstrate that the unsanctioned SIF by VANDU has saved taxpayers’ money, and should be expanded to prevent further HCV and HIV cases.

## Discussion

This analysis reviewed and utilized a cost-benefit/cost-effectiveness analysis in order to determine whether operating an unsanctioned SIF, where peers were actively engaged in operating the facility and were able to offer assisted injection, was an efficient use of public health resources. The value of averted HIV and HCV cases far outweighs the cost of SIFs and the equipment employed. Moreover, additional unsanctioned SIFs operated by peer-drug users would be a good value for the resources they consume. In fact, the results here suggest that additional peer-run SIFs that can offer assisted injection will benefit the publically funded health care system, even more than other potential cost-savings such as the prevention of endocarditis, overdose deaths, subcutaneous abscesses, cellulitis, and soft-tissue infections.

Nevertheless, it should be noted that this study did not rely on a more complex method that considers quality-adjusted life years (QALYs). In effect, the methods and models employed in this study may be considered rudimentary in comparison to more complex models used previously by Bayoumi and Zaric [5] or Bayoumi *et al.* [6] in the area of costing studies of SIF. This model did not formally take into account the dynamics of the social system and a score of parameters such as increase in detoxifications, methadone treatments, or secondary HIV transmission through sexual contact. Rather, it employed static mathematical models that only consider the reduction in HIV and HCV cases linearly. Such models are typically used to evaluate needle exchange programs [24, 34].

Moreover, in this study, the medical cost of incident HIV has significantly underestimated the cost of treatment, diagnosis, clinical care, and lifelong provisions of medication such as ART. Furthermore, the cost of hospitalizations for HIV-related complications have also been significantly underestimated in the current study since PWID are commonly known to end up using hospital services because of the complications related to HIV [53, 64]. Consequently, the estimated lifetime healthcare costs of an HIV diagnosis is nearly double the estimate presented in this research (*e.g.*, US$326,500 or CAN$409,725) [53, 64].

These models have been previously adapted to the context of SIFs by a number of peer-reviewed studies [3, 4, 27, 28, 30, 31, 33, 60, 61]. They have proven to be instructive and realistic, generating results that have been verified with known occurrences of HIV and HCV. For example, Jozaghi et al’s [33] analysis of establishing SIFs in Ottawa estimated approximately the same amount of HIV and HCV infections, in addition to relying on calculations made by other authors, such as Bayoumi *et al.* [6], that have used more complex models that consider QALYs. In effect, in some instances, the complex models based on QALYs have been criticized for over estimating the number of prevented cases of HIV and HCV [10].

The success rates of such programs, according to Jozaghi [29] and Des Jarlais *et al.* [10]), is based on locating SIFs where drug users live and in settings with high rates of public drug use. The current SIF, known to many Canadians as Insite, despite various restrictions, has proven effective in reducing injections in public, while lowering the rate of fatalities caused by overdose and infectious diseases [35, 36, 48, 74]. Moreover, Insite has not resulted in increased rates of crime, drug dealing, public injection, public syringe disposal, or public disorder around its vicinity [11, 16, 66]. Therefore, such benefits as the one noted above, could potentially be expanded with additional SIFs that could accommodate various PWID.

In summary, this study, through the use of two mathematical models, has shown that the former unsanctioned SIF in Vancouver was extremely cost-effective. The models in this paper are similar to those used by Andresen and Boyd [3], Andresen and Boyd [3]) and Jozaghi *et al.* [32]), Jozaghi *et al.* [33] and Jozaghi [27, 28]), which have demonstrated the cost-effectiveness of SIFs and supervised smoking facilities in various Canadian settings. This paper shows that the number of new HIV and HCV infections averted, and the associated benefits and cost-effectiveness, are more than enough to cover the cost of operating more than one SIF in Vancouver that could potentially be operated by peers who can offer assisted injection. As result, the findings of this costing study and the Qualitative study by McNeil *et al.* [51] have shown the critical role that unsanctioned SIFs, despite not having a license to operate legally, could play in reducing blood-borne infections and saving health care costs

Ultimately, the closure of the small facility by VANDU, a consequence of a threat by their funding agency, Vancouver Coastal Health, has been problematic, potentially placing many PWID at increased risk of contracting HIV and HCV infections. The concept of a peer-driven unsanctioned SIF, where drug users can assist their peers inject, could be relevant to many U.S. cities such as San Francisco, Baltimore and New York that are facing health concerns attributed to injection drug use. An unsanctioned SIF in the noted US cities would increase access to lifesaving services and would serve to restore some dignity and respect to the lives of many drug users who are placed at increased risk because of their relapsing medical condition.
